# Neuroprotective Metabolites of *Hericium erinaceus* Promote Neuro-Healthy Aging

**DOI:** 10.3390/ijms22126379

**Published:** 2021-06-15

**Authors:** Elisa Roda, Erica Cecilia Priori, Daniela Ratto, Fabrizio De Luca, Carmine Di Iorio, Paola Angelone, Carlo Alessandro Locatelli, Anthea Desiderio, Lorenzo Goppa, Elena Savino, Maria Grazia Bottone, Paola Rossi

**Affiliations:** 1 Laboratory of Clinical & Experimental Toxicology, Pavia Poison Centre, National Toxicology Information Centre, Toxicology Unit, Istituti Clinici Scientifici Maugeri IRCCS, 27100 Pavia, Italy; elisa.roda@icsmaugeri.it (E.R.); carlo.locatelli@icsmaugeri.it (C.A.L.); 2 Department of Biology and Biotechnology “L. Spallanzani”, University of Pavia, 27100 Pavia, Italy; ericacecilia.priori01@universitadipavia.it (E.C.P.); daniela.ratto01@universitadipavia.it (D.R.); fabrizio.deluca01@universitadipavia.it (F.D.L.); carmine.diiorio01@universitadipavia.it (C.D.I.); paola.angelone01@universitadipavia.it (P.A.); mariagrazia.bottone@unipv.it (M.G.B.); 3 Department of Earth and Environmental Science, University of Pavia, 27100 Pavia, Italy; anthea.desiderio01@universitadipavia.it (A.D.); lorenzo.goppa01@universitadipavia.it (L.G.); elena.savino@unipv.it (E.S.)

**Keywords:** healthy aging, frailty, cerebellum, *inflammaging*, oxidative stress, ergothioneine, *Hericium erinaceus*, neuroprotection

## Abstract

Frailty is a geriatric syndrome associated with both locomotor and cognitive decline, typically linked to chronic systemic inflammation, i.e., *inflammaging*. In the current study, we investigated the effect of a two-month oral supplementation with standardized extracts of *H. erinaceus*, containing a known amount of Erinacine A, Hericenone C, Hericenone D, and L-ergothioneine, on locomotor frailty and cerebellum of aged mice. Locomotor performances were monitored comparing healthy aging and frail mice. Cerebellar volume and cytoarchitecture, together with inflammatory and oxidative stress pathways, were assessed focusing on senescent frail animals. *H. erinaceus* partially recovered the aged-related decline of locomotor performances. Histopathological analyses paralleled by immunocytochemical evaluation of specific molecules strengthened the neuroprotective role of *H. erinaceus* able to ameliorate cerebellar alterations, i.e., milder volume reduction, slighter molecular layer thickness decrease and minor percentage of shrunken Purkinje neurons, also diminishing inflammation and oxidative stress in frail mice while increasing a key longevity regulator and a neuroprotective molecule. Thus, our present findings demonstrated the efficacy of a non-pharmacological approach, based on the dietary supplementation using *H. erinaceus* extract, which represent a promising adjuvant therapy to be associated with conventional geriatric treatments.

## 1. Introduction

Aging is a universal process characterized by a gradual decline in physical and cognitive functions. As age increases, a variety of changes occur, including brain atrophy, oxidative stress, and reduced antioxidant mechanisms, contributing to impairment of cognitive and locomotor performances [1].

The term frailty was proposed to describe a multisystemic impairment scenario, which negatively affects the health of an elderly individual, contributing to aggravate a clinical condition that is often already compromised. Recently, the World Health Organization (WHO) recognized frailty as an increasingly widespread syndrome that should be prevented, reversed, or at least mitigated to improve the quality of life in the elderly [2].

Frailty is a complex geriatric syndrome characterized by age-associated declines in physiologic reserve and functions through multiorgan systems, leading to enhanced vulnerability for adverse health outcomes [3,4]. Compelling evidence linked frailty to both immunosenescence and chronic systemic inflammation, the so-called *inflammaging* [5,6,7]. This latter mechanism is a typical biomarker of accelerated aging, being also a risk factor for cardiovascular diseases, cancer, dementia, cognitive decline, and physical disabilities [7,8]. In addition, *inflammaging* increases susceptibility to stress-related molecules [6,7].

Several works demonstrated that locomotor frailty predicts cognitive impairment and dementia during aging [9,10,11,12], and, in particular, the locomotor decline seems to be a risk factor of future cognitive deterioration [13,14]. In this scenario, the prevention, detection and the reversion of physical frailty is imperative, aimed at avoiding neurodegenerative diseases pathogenesis and cognitive impairment outcomes.

An imbalance in the REDOX system is critical not only in aging but even more in frail subjects. The accumulation of reactive oxygen and nitrogen species (RONS) during aging induces cellular damages and contributes to the onset of age-associated tissue impairment. Based on this evidence, the oxidative stress theory of aging was formulated and, over the years, the role of oxidative stress in the worsening of age-related diseases has been consolidated [15]. A redox imbalance is particularly critical in frail subjects during aging. Furthermore, oxidative stress induces the secretion of proinflammatory molecules and chemokines that promote protein degradation and contribute to cellular degeneration [16].

The brain is very sensitive to oxidative stress-induced damage, and the overproduction of free radicals during aging is suggested to be responsible for age-associated brain structural corrosion and functional decline [1]. Experimental and clinical evidence supported that age-related brain atrophy could worsen locomotor and cognitive performances [1]. Specifically, aged-derived increase of oxidative stress leads to cellular damages in the cerebellum, e.g., gray matter volume reduction and atrophy [17].

Furthermore, the morphological signs observed in cerebellum have been correlated with slow walking speed and reduction in both physical and social behaviors suggesting that frailty could promote neuronal degeneration in elderly patients [12,18]. Notably, cerebellar Purkinje neurons are vulnerable to aging, displaying considerable alterations in both morphology and function, e.g., cell number reduction and decrease in synapse density [19].

*Hericium erinaceus* is an edible and medicinal mushroom and it seems to stand out as an exceptional health-promoting species. Available generally in North temperate latitudes (including Italy), *H. erinaceus* can be identified by its long spines, for its appearance on hardwoods and its tendency to grow with a single tuft of dangling spines. Given its shape, it is also known as Lion’s mane and Monkey Head Mushroom [20,21]. *H. erinaceus* is able to regulate cytokines and mitogen-activated protein kinases expressions, and transcription factors at the molecular level: *H. erinaceus* performs medicinal activities at tissue, organ, and organism levels. Indeed, *H. erinaceus* synthesizes at least 70 different bioactive metabolites, such as β-glucans, erinacines, hericenones, alkaloids, sterols, and volatile aroma compounds [21]. Thanks to these biological compounds, *H. erinaceus* exerts many health-promoting properties [21], such as antibiotic [22,23], anticancer [24,25], antioxidant [26,27], antifatigue [28], antisenescence [14,29], neuroprotective [30,31,32], antidepressive, and antianxiety [33,34] activities.

Furthermore, both in *H. erinaceus* fruiting body and mycelium ergothioneine is found [35]. L-ergothioneine is a water-soluble thiol that can only be absorbed from the diet because animals and plants cannot synthesize this compound, produced solely by bacteria and mushrooms. L-ergothioneine displays antioxidant and cytoprotective capabilities [36,37]. An increasing number of scientific articles demonstrated the potential of L-ergothioneine as therapy for several diseases, such as preeclampsia [38,39], neurodegenerative [40,41,42,43], cardiovascular [44,45], and endothelial-muscular [46,47,48] pathologies.

Based on this knowledge, *H. erinaceus* appears an excellent candidate to prepare novel mushroom-based pharmaceuticals/medicines and functional foods [20]. Nevertheless, the standardization of medicinal mushrooms-derived dietary supplements is still in development, since proper standards and protocols have yet to be identified [49].

Our previous findings demonstrated the neuroprotective action and nootropic effect of *H. erinaceus* in adult wild-type mice. Specifically, dietary supplementation with *H. erinaceus* was effective at (i) increasing hippocampal neurotransmission, locomotor performances and recognition memory in wild-type mice [50,51], and (ii) improving recognition memory in frail mice during aging, also inducing hippocampal and cerebellar neurogenesis [14].

In the current study, we investigated the effect of a two-month oral supplementation with standardized extracts of *H. erinaceus* (He1), containing a known amount of Erinacine A, Hericenone C, Hericenone D, and L-ergothioneine, on locomotor frailty and cerebellum of aged mice. We decided to supplement only frail mice, then compare them to healthy aged animals to explore the potential occurrence of a recovery process, in which *H. erinaceus*-supplemented frail mice could have reverted, at least in part, to healthy aging.

Specifically, we monitored the locomotor performances comparing animals belonging to the two experimental groups (healthy aged vs. frail mice), and, further, we evaluated the cerebellar volume and cytoarchitecture, together with inflammatory and oxidative stress pathways, jointly with a neuroprotective molecule and a key longevity regulator, focusing on senescent frail animals.

## 2. Results

### 2.1. Array of Metabolites in Hericium erinaceus Extract

Italian *Hericium erinaceus* (He1) was collected in Siena province (Tuscany, Italy) and identified on the macro- and micro-morphological characteristics of the species. Culturing and extraction procedures are meticulously reported in Materials and Methods section (see Section 4.3).

Using HPLC-UV-ESI/MS, and by comparison with standard solutions, the presence and amount of different metabolites, i.e., Erinacine A, Hericenone C, Hericenone D was identified and quantified [14,52]. Specifically, He1 extract contained 150 µg/g Erinacine A, 500 µg/g Hericenone C, and about 20 µg/g Hericenone D.

**Figure 1:**
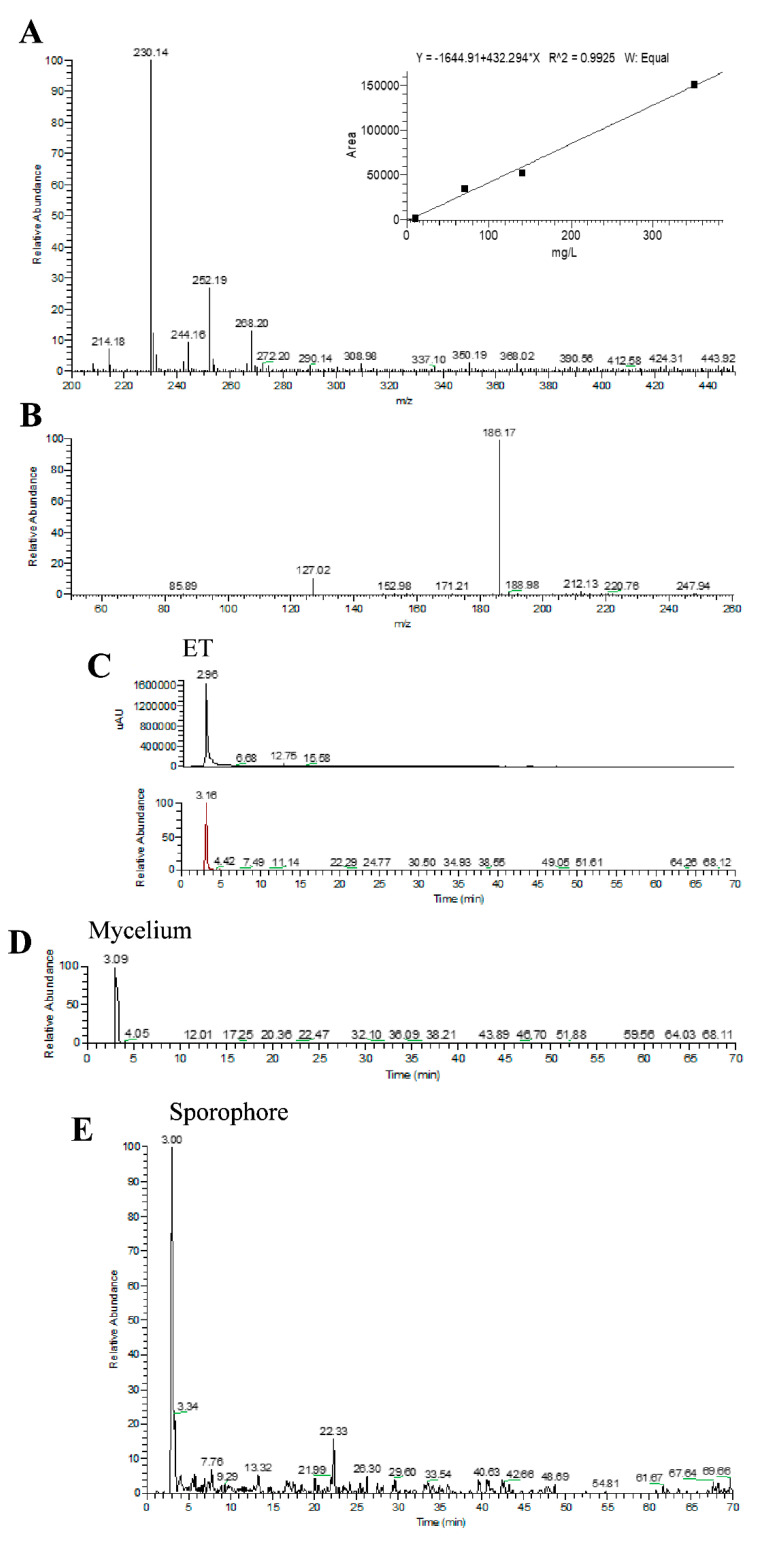
HPLC-UV-ESI/MS was used for quantifying the Ergothioneine (ET) amount in He1 extracts. (**A**) ESI/MS spectrum of ET. Panel (top, right) reports calibration curve and linear regression curve for ET. (**B**) ESI-MS/MS spectrum of ET obtained by fragmentation of ion *m*/*z* 230 shows two fragment ions *m*/*z* 186 and *m*/*z* 127. (**C**) Standard molecule of L-(+)-ET at 70 ppm: UV trace (top) and mass spectrum (MS)/MS Selected Reaction Monitoring *m*/*z* 230 > *m*/*z* 186 trace (bottom). (**D**) MS/MS Selected Reaction Monitoring *m*/*z* 230 > *m*/*z* 186 trace of He1 mycelium. (**E**) MS/MS Selected Reaction Monitoring *m*/*z* 230 > *m*/*z* 186 trace of He1 WT sporophore.

In addition, the amount of ergothioneine (ET) in mycelium and sporophore extracts of He1 was measured. The ET calibration curve was constructed with concentrations ranging from 10 to 350 µg/mL (see Section 4). Linear least-square regression analysis for the calibration curve showed correlation coefficient of 0.9925 with respect to the peak area (Figure 1, Panel A, top right insert), demonstrating a good linear relationship in the different ranges tested.

In particular, ET was identified by comparing retention time and ESI/MS-MS spectrum with the authentic standard. Typical ions of ET in the ESI/MS spectrum (Figure 1, Panel A) are hydrogen, sodium and potassium adducts (Table 1). ESI-MS/MS spectrum of ET (Figure 1, Panel B) obtained by fragmentation of ion *m*/*z* 230 shows two fragment ions *m*/*z* 186 and *m*/*z* 127 (Table 1). To obtain better sensitivity, ET was then quantified by multiple-reaction-monitoring (MRM) transition *m*/*z* 230 > 127.

Figure 1 panel C displays the traces regarding the standard molecule of ET at 70 ppm. On the top, the figure shows the UV trace and, on the bottom, it represents the mass spectrum (MS) trace (MS/MS Selected Reaction Monitoring *m*/*z* 230 > *m*/*z* 186 traces).

Figure 1 panel D displays the mass spectrometry (MS) chromatographic traces of He1 lyophilized mycelium. Notably, both ESI-MS/MS spectrum of the standard molecule of ET at 70 ppm (retention time at 3.16 min; Figure 1, Panel C) and the He1 mycelium spectrum showed the peak of ET (retention time at 3.09; Figure 1, Panel D). By comparison with the calibration curve, we measured the content of ET in He1 lyophilized mycelium. It should be noted that the ET content of 580 µg/g is slightly more compared to data reported in literature (0.4 mg/g reported by Chen et al. [53]).

Figure 1 panel E shows the mass spectrometry (MS) chromatographic traces of the He1 sporophore. The content of ET (retention time at 3.00) in the He1 WT sporophore was 0.34 mg/g (d.w.), measured by comparison with ET calibration curve. This value is slightly smaller compared to data reported in literature, i.e., 0.96 mg/g [54] and 1.12 mg/g [55].

### 2.2. H. erinaceus Reverted the Locomotor Decline during Senescence in Wild-Type Mice

During senescence, the mean speed and resting time were recognized as the most sensitive locomotor parameters, decreasing with aging, while the maximum speed (cm/s) did not change with frailty [14].

Therefore, locomotor performances were measured by the mean speed (cm/s) and the resting time (s) in untreated (n = 8) versus He1 treated mice (n = 7) at 21.5 (T1) and 23.5 (T2) months of age. Locomotor frailty index (FI) was obtained by averaging the mean speed and the resting time FIs and its measurement at T1 was the selection criteria for dividing the mice in two groups: non-frail and frail mice (see Section 4).

**Figure 2:**
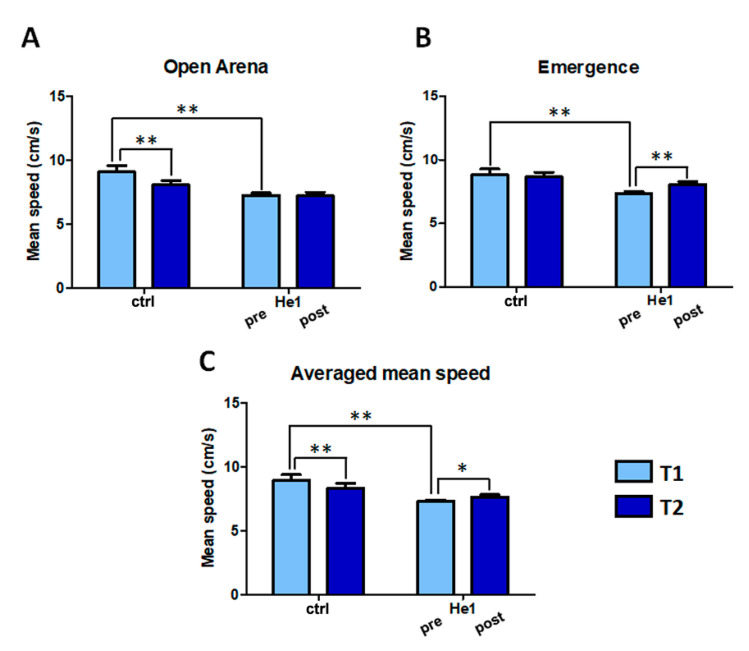
*H. erinaceus* treatment reverted the mean speed decline during senescence. Mean speed (cm/s) estimated during open arena test (Panel **A**) and emergence task (Panel **B**). Panel **C**: Averaged mean speed, calculated between mean speed obtained in open arena and emergence tests in untreated and He1 treated mice before (T1) and after (T2) treatment. Two-way ANOVA was performed for statistical analysis: * *p* < 0.05; ** *p* < 0.01.

In untreated mice, the mean speed decreased significantly during aging of 11.4% in the open arena test (Figure 2, Panel A), and a similar nonsignificant trend was observed in the emergence test (Figure 2, Panel B). Before *H. erinaceus* treatment, at T1, frail mice displayed a significant decrease of the mean speed compared to untreated mice by 20% in the open arena and by 16.9% in the emergence test. After *H. erinaceus* treatment this decline remained unchanged in the open arena test and significantly improved in the emergence test (Figure 2, Panels A and B). Overall, the averaged mean speed (from open arena and emergence test) significantly decreased during senescence in untreated mice from T1 to T2, whereas significantly increased after He1 treatment in frail mice (Figure 2, Panel C). Notably, whereas the untreated and pre-He1 treated mice showed significant differences at T1 in both open arena and emergence test, the treatment brought mice back to comparable levels at T2.

**Figure 3:**
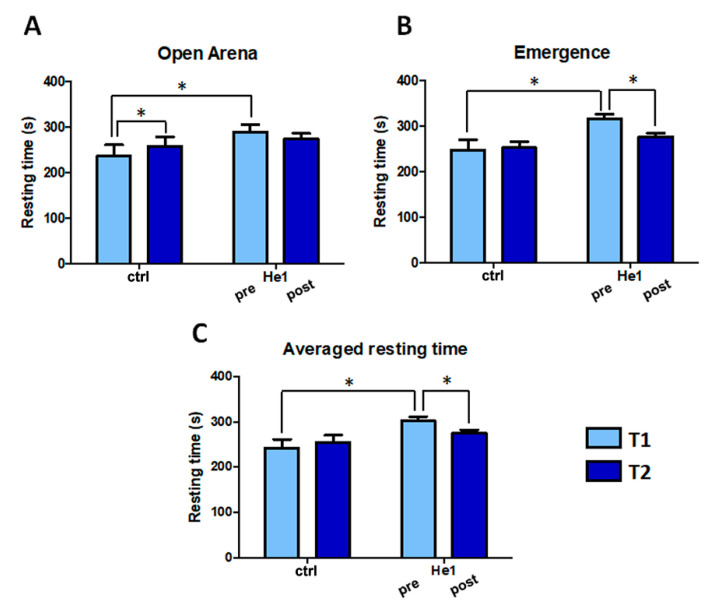
*H. erinaceus* treatment reverted the resting time increase during aging: mean resting time (s) estimated during open arena (Panel **A**) and emergence tasks (Panel **B**). Panel **C**: Averaged resting time obtained by both tasks, in untreated (ctrl) and He1 treated mice (He1) before (T1) and after (T2) treatment. Two-way ANOVA was performed for statistical analysis: * *p* < 0.05.

In untreated mice, in the open arena test, the resting time significantly increased by 9.4%, and a nonstatistically significant difference was observed in the emergence test. Before He1 treatment, at T1, frail mice displayed a significant increase of resting time compared to untreated mice by 18.16% in the open arena and by 21.74% in the emergence test. Interestingly, at T2 He1 treatment reverted the increase in resting time recorded in untreated mice between T1 and T2, caused by the ageing process. In particular, the resting time in He1 treated mice at T2 slightly decreased in the open arena (Figure 3, Panel A), and decreased 12.44% in the emergence test, reaching the statistical significance (Figure 3, Panel B). The averaged resting time (from open arena and emergence test) significantly increased during senescence in untreated mice, whereas it significantly decreased, by about 9%, in He1 treated mice (Figure 3, Panel C). Notably, whereas the untreated and He1 treated mice showed significant differences at T1, the treatment brought mice back to comparable levels at T2.

Finally, we investigated the effects of physiological aging and *H. erinaceus* supplementation by tuning an overall locomotor FI.

**Figure 4:**
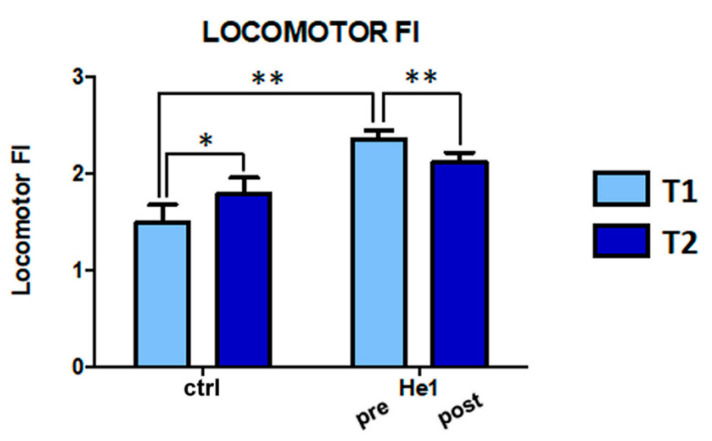
Locomotor frailty index in untreated and He1 treated mice at T1 and T2. Two-way ANOVA was performed for statistical analysis: * *p* > 0.05; ** *p* < 0.01.

At T1, the overall locomotor FI of the frail mice (pre-He1 treated animals) was 51% higher than that measured in untreated mice (2.36 ± 0.07 vs. 1.63 ± 0.21, Figure 4). Interestingly, the *H. erinaceus* treatment significantly decreased by about 10% of the overall locomotor frailty index (2.36 ± 0.07 at T1 vs. 2.12 ± 0.09 at T2), whereas it significantly increased between T1 and T2 in untreated mice (1.63 ± 0.21 at T1 vs. 1.79 ± 0.17 at T2). Notably, whereas the pre-He1 treated mice were significantly frailer compared to untreated animals at T1, the treatment brought mice back to comparable levels, with no significant differences in the locomotor frailty index at T2 between the two experimental groups (Figure 4).

### 2.3. H. erinaceus Effect on Cerebellar Cytoarchitecture, Neuroinflammation and Oxidative Stress Pathway

Histochemical and immunohistochemical reactions were conducted on sagittal sections of the cerebellar vermis from both untreated (Ctrl) and *Hericium*-treated mice (He1) at T2 (23,5-month-old mice). The evaluations were performed on the posterior region, specifically on the neocerebellar lobules VI–VIII, known to be particularly impacted by age and correlated with general cognitive and motor function [56,57,58,59].

#### 2.3.1. H&E and Nissl Staining

We evaluated the cerebellar volume together with morphological characteristics of cerebellar cortex, exploiting potential cytoarchitecture alterations, comparing senescent untreated animals with He1-supplemented aged mice, using Haematoxylin and Eosin (H&E) and Nissl staining.

**Figure 5:**
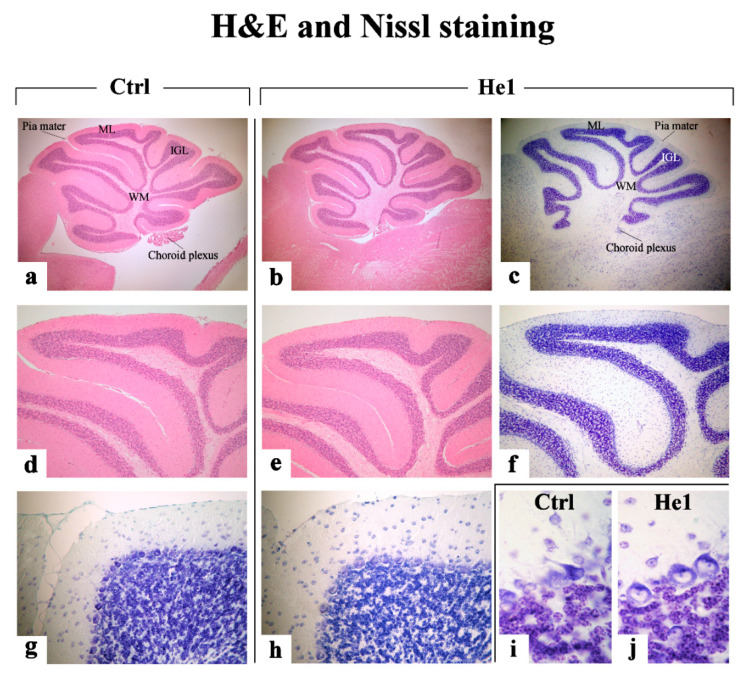

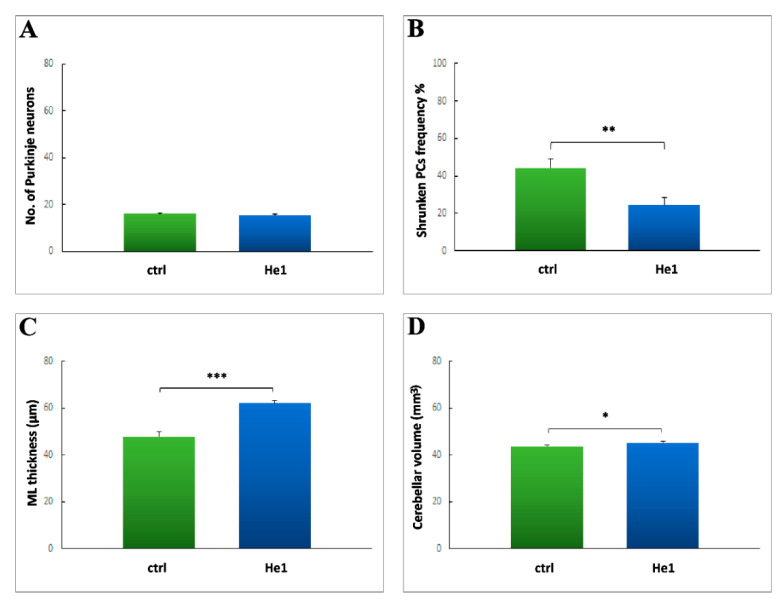
Representative H&E and Nissl-stained cerebellar specimens, in untreated (**a**,**d**,**g**,**i**) and He1-treated mice (**b**,**c**,**e**,**f**,**h**,**j**). Light microscopy magnification: 20× (**a**–**c**); 40× (**d**–**h**); 100× (**i**,**j**). Panels (**A**–**D**) Histograms showing the quantitative measurement of the whole number of PCs, the percentage of shrunken PCs, the ML width and the entire cerebellar volume, respectively. *p* values calculated by unpaired Student’s *t*-test: * *p* < 0.05; ** *p* < 0.01; *** *p* < 0.001.

Similarly, H&E and Nissl results (Figure 5) demonstrated a well-preserved physiological cerebellar cytoarchitecture in both groups, displaying the typical three-layered cortical organization, i.e., molecular, Purkinje, and granular layers (ML, PCs, and IGL, respectively, from the outer to inner one). The ML was formed of few small cells together with numerous fibers. The large pyriform PCs appeared regularly arranged in a single row along the outer margin of the IGL with vesicular nuclei. The IGL showed tightly packed small rounded cells with deeply stained nuclei. Neither malformation or supernumerary extroversions, nor PCs loss (total number of PCs) were detected (Figure 5, Panel A).

Nonetheless, comparing animals from the two experimental groups, age-related changes were determined in untreated mice only, in which (i) the volume of the entire cerebellum was significantly reduced (43.73 ± 0.44 mm^3^), (ii) about 44% of the total PCs appeared evidently shrunken, and, parallelly, (iii) a significant reduction of the ML thickness (47.81 ± 2.23 µM) was measured. Notably, He1 supplementation seemed to play a significant protective effect since He1 animals exhibited a bigger cerebellar size (45.35 ± 0.39 mm^3^), only 24% of the total PCs was characterized by an altered morphology and the ML was greater in width (62.02 ± 0.98 µM) (Figure 5, Panel C, respectively).

#### 2.3.2. Picrosirius Red Staining: Fibrillar Collagen Networks Evaluation

**Figure 6:**
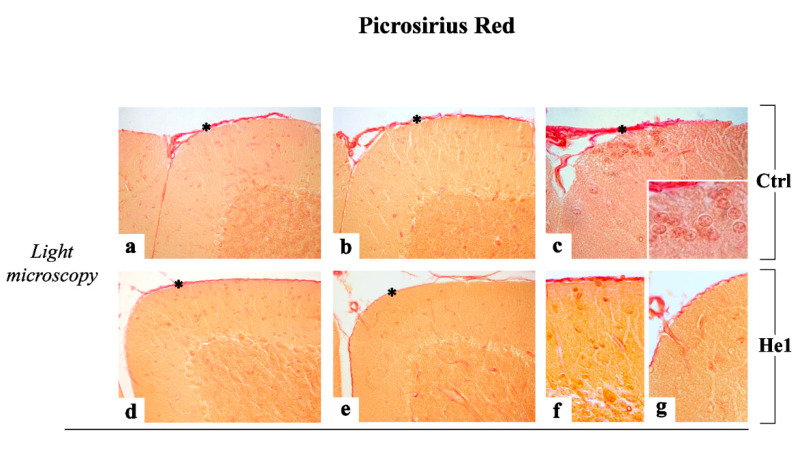

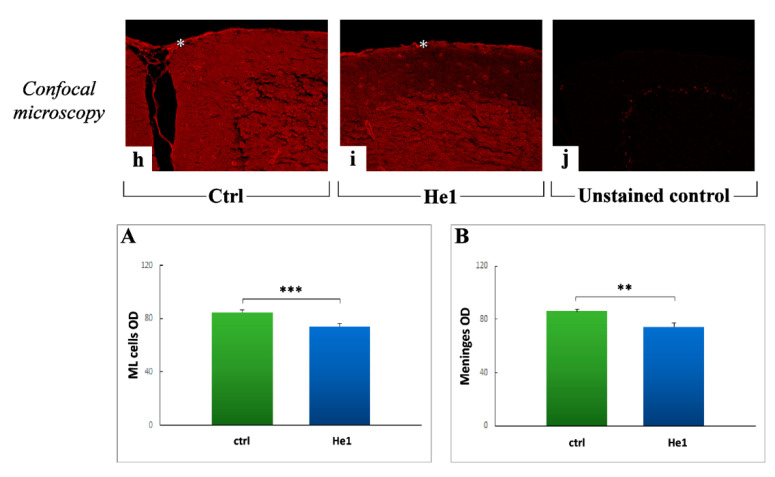
Representative PSR-stained cerebellar specimens, in untreated (**a**–**c**,**h**,**j**) and He1-treated mice (**d**–**g**,**i**) investigated by both light and confocal microscopy. Magnification: 40× (**a**–**e**,**h**–**j**); 100× (insert in **c**,**f**,**g**). Panels (**A**,**B**) Histograms showing the quantitative analysis of histochemically positive ML cells and meningeal fibers OD, respectively. *p* values calculated by unpaired Student’s *t*-test: (**) < 0.01, and (***) < 0.001. Asterisks in **a**–**e**,**h**–**i**: meningeal fibers.

Picrosirius Red (PSR) staining was employed as the most sensitive tool to appraise collagen networks in paraffin-embedded tissue sections. In both experimental groups, i.e., untreated and He1 mice, collagen fibers were evidently localized in the meninges (Figure 6). Notably, some clusters of histochemically positive cells were observed in the upper external part of the molecular layer (ML) in untreated animals only (Figure 6, insert in c), while both He1 and untreated mice displayed PSR positive basal laminae of the cerebellar capillaries.

Notably, in the ML (Figure 6, Panel A) as well as at pial surface level (Figure 6, Panel B), the quantitative analysis evidenced a significant decrease of collagen fibers optical density (OD) in He1 mice compared to untreated animals (74.22 ± 2.15 vs. 84.71 ± 1.82, *p* < 0.001 in GCs and 74.39 ± 2.89 vs. 85.91 ± 1.79, *p* < 0.01, in meninges, respectively). These data obtained using bright field microscopy were further confirmed by confocal microscopy observation.

#### 2.3.3. Inflammatory Pathway (IL6, GFAP) and HIF1α Assessment

Based on literature evidence supporting the role played by immune system in influencing brain plasticity phenomena and occurrence of age-dependent enhanced neuroinflammation, we first performed an immunohistochemical evaluation of the presence/distribution of the pro-inflammatory cytokine Interleukin-6 (IL6), as specific markers of inflammatory pathway.

**Figure 7:**
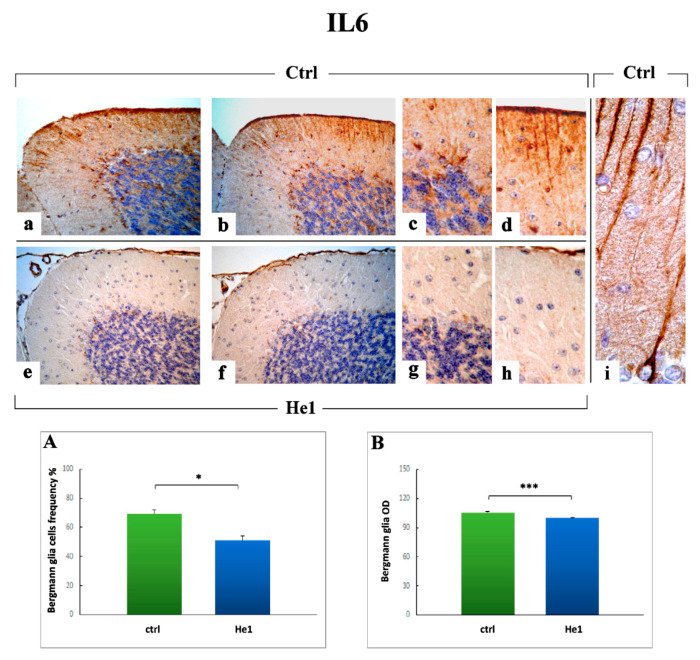
IL6 DAB-immunostaining reaction in untreated (**a**–**d**,**i**) and He1-treated mice (**e**–**h**). Light microscopy magnification: 40× (**a**,**b**,**e**,**f**); 100× (**c**,**d**,**g**,**h**). Panels (**A**,**B**) Histograms showing immunopositive BG cells frequency and OD, respectively. *p* values calculated by unpaired Student’s *t*-test: * *p* < 0.05, *** *p* < 0.001.

The IL6 immunopositivity was mainly observed in numerous cells localized closed to the Purkinje neurons soma, both in untreated mice and He1 animals (Figure 7a,c). Notably, the presence of numerous thin IL6-positive fibers, which ran parallel in the thickness of molecular layer (ML) reaching the pial surface, was only detected in untreated animals (Figure 7b). These fibers, appearing more markedly stained in the upper part of this layer, showed a regular feature. The observed IL6 immunopositive cells and fibers could be reasonably considered as the main constituents of the Bergmann glia (BG), with astrocytic cell bodies around the Purkinje cells and the polarized processes, i.e., radial fibers, extending over the full depth of the ML (Figure 7i). The quantitative analysis of IL6 immunopositive BG cells frequency (50.92 ± 3.23 vs. 69.24 ± 2.60, *p* < 0.05) and OD (100.02 ± 0.31 vs. 105.56 ± 0.72, *p* < 0.001) (Figure 7, Panels A and B, respectively) revealed a significant decreased in He1 mice compared to untreated animals.

**Figure 8:**
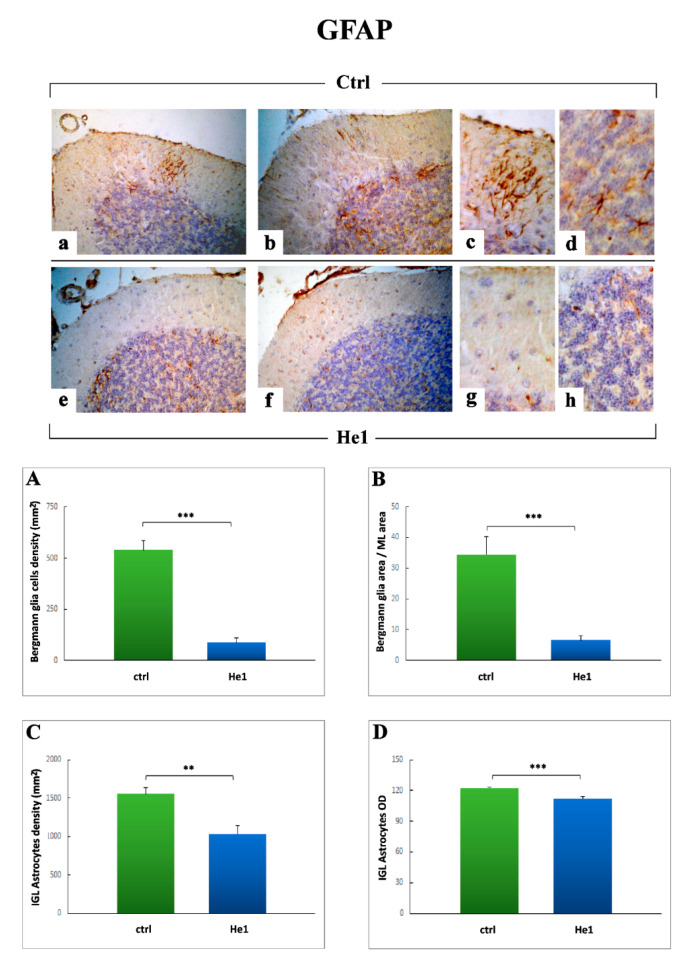
GFAP DAB-immunostaining reaction in untreated (**a**–**d**) and He1-treated mice (**e**–**h**). Light microscopy magnification: 40× (**a**,**b**,**e**,**f**); 100× (**c**,**d**,**g**,**h**). Panels (**A**–**D**) Histograms showing quantitative analyses of BG immunopositive cell density, ratio between GFAP-immunopositive area in the ML and whole ML area, IGL immunopositive astrocytes density and OD, respectively. *p* values calculated by unpaired Student’s *t*-test: ** *p* < 0.01, *** *p* < 0.001.

As a second step, based on the notion that a reactive gliosis could be a direct consequence of IL6 overexpression [60,61], we assessed the presence/distribution of Glial fibrillary acidic protein (GFAP), as a specific molecular marker of the Bergmann glia (Figure 8). The GFAP immunoreactivity was markedly evident in untreated animals (Figure 8a–d), in which manifest GFAP-positive glial fibers were detected. These fibers appeared sometimes thickened and twisted, with an irregular course in the thickness of ML, being frequently more marked in some area of this layer. Several GFAP-positive fibers often displayed a thickened and intensely stained end-feet in the pial surface of the ML. Moreover, a significant number of GFAP-immunopositive IGL astrocytes were observed. Differently, an almost complete absence of immunonopositive fibers was detected in the ML of He1 mice, in which rare IGL immunoreactive astrocytes were also observed (Figure 8e–h).

The quantitative analyses confirmed a significant decrease in both GFAP immunopositive fibers and astrocytes density (Figure 8, Panels A and C) and OD (Figure 8, Panels B and D), respectively, in He1 mice compared to untreated animals, according to the previous resulted obtained after IL6 staining. Specifically, the following measurements were achieved comparing untreated to He1 mice: GFAP-immunopositive BG cells density (87.61 ± 22.75 vs. 539.95 ± 46.57, *p* < 0.001); GFAP immunopositive BG area/ML area (6.53 ± 1.53 vs. 34.49 ± 5.68, *p* < 0.001); GFAP immunoreactive IGL astrocytes density (1033.42 ± 110.54 vs. 1557.39 ± 80.76, *p* < 0. 01); GFAP immunoreactive astrocytes OD (112.14 ± 1.90 vs. 122.28 ± 0.68, *p* < 0.001).

To integrate the study of the inflammatory pathway, based on the notion that hypoxia-inducible factors (HIFs) play as essential regulators of inflammation [62,63], we investigated the expression of HIF1α, which is regulated at protein level in an oxygen-sensitive manner, and is known as a critical transcription factor with an essential role in aging-related pathology [64]. Moreover, since angiogenic cytokines may upregulate vascular endothelial factor (VEGF), whose expression is also directly induced by HIF1α, we additionally examined VEGF, as a peculiar molecule known for its wide-ranging functions, known for its role in angiogenesis and vasculogenesis, to promote vascular development, permeability, and endothelial outspreading, but also investigated based on its role as a neurogenic, neurotrophic and neuroprotective factor in the nervous system [65].

**Figure 9:**
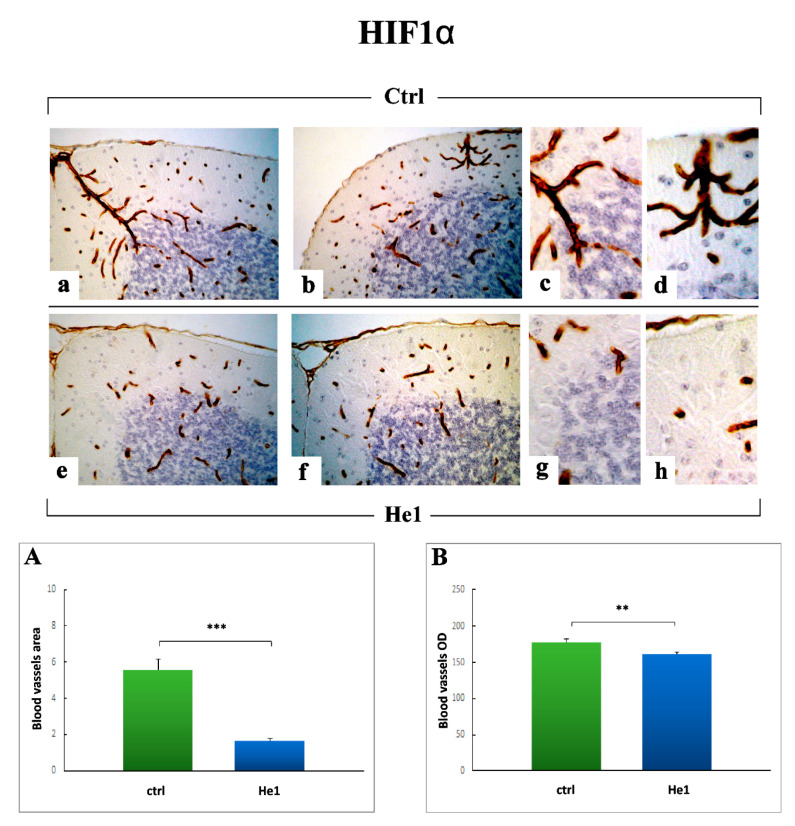
HIF1α DAB-immunostaining reaction in untreated (**a**–**d**) and He1-treated mice (**e**–**h**). Light microscopy magnification: 40× (**a**,**b**,**e**,**f**); 100× (**c**,**d**,**g**,**h**). Panels (**A**,**B**) Histograms showing HIF1α-immunopositive blood vessels area and OD, respectively. *p* values calculated by unpaired Student’s *t*-test: ** *p* < 0.05, *** *p* < 0.001.

The HIF1α immunopositivity was clearly detected in blood vessels, i.e., vascular endothelial cells, localized in the whole width of the cerebellar cortex, both in untreated animals and He1 mice (Figure 9). In fact, the presence of widespread HIF1α-positive vessels, mainly localized in the thickness of ML and characterized by the presence of several convoluted ramifications, was detected (Figure 9). These vessels, markedly stained, appeared particularly numerous and extended in untreated animals compared to He1 mice. The subsequent quantitative analysis supported a significant decrease of both blood vessels area and OD in He1 mice compared to untreated ones (Figure 9, Panels A and B; 1.65 ± 0.15 vs. 5.60 ± 0.59, *p* < 0.001 and 161.66 ± 3.16 vs. 178.21 ± 3.57, *p* < 0.01, for area and OD, respectively).

**Figure 10:**
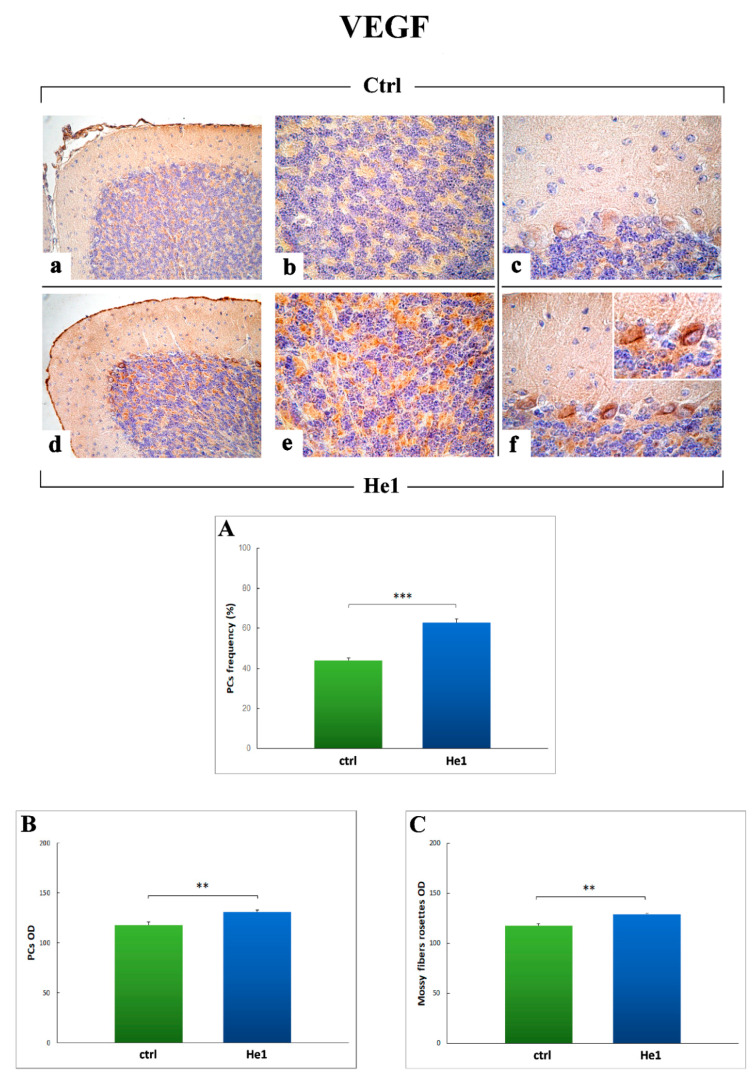
VEGF DAB-immunostaining reaction in untreated (**a**–**c**) and He1-treated mice (**d**–**f**). Light microscopy magnification: 40× (**a**–**f**); 100× (*insert* in **f**). Panels (**A**–**C**) Histograms showing the quantitative measurement of VEGF-immunoreactive cell frequency and OD for both Purkinje neurons and mossy fibers rosettes. *p* values calculated by unpaired Student’s *t*-test: ** *p* < 0.01, *** *p* < 0.001.

Concerning VEGF (Figure 10), the immunopositivity appeared evidently localized in the sizeable soma of Purkinje neurons, evidently more marked in He1 mice (Figure 10d,f) compared to untreated animals (Figure 10a,c). Showing a similar trend (i) a striking immunoreactivity was also detected in the large mossy fiber rosettes located in the IGL (Figure 10b,e). Notably, the meningeal formation at the pial surface was also significantly immunomarked (Figure 10d). Any immunopositivity was observed in cerebellar cortex blood vessels neither in He1 mice nor in untreated animals (Figure 10a–f).

VEGF-immunoreactivity, quantitatively measured in terms of immunopositive PCs cell frequency, significantly increased in He1 treated mice compared to untreated animals (62.80 ± 1.76 vs. 44.10 ± 1.22, *p* < 0.001, respectively) (Figure 10, Panel A). As well, the assessment of VEGF-immunoreactivity OD, evaluated both at PCs soma and mossy fiber rosettes levels, significantly increased in He1 mice compared to untreated ones (130.94 ± 1.82 vs. 117.93 ± 0.77, *p* < 0.01 and 128.74 ± 0.96 vs. 117.62 ± 1.82, *p* < 0.01, respectively) (Figure 10, Panels B and C).

#### 2.3.4. Oxidative Stress Pathway: SOD1, NOS2 and COX2 Immunohistochemical Assessment

Literature evidence highlighted oxidative damage involvement in aging and age-associated cognitive impairment as a consequence of an increased reactive oxygen species (ROS) production and/or a decrease in antioxidant scavengers [66]. We assessed by immunohistochemistry the presence/distribution of Superoxide Dismutase 1 (SOD1), Nitric oxide synthase 2 (NOS2) and Cyclooxygenase 2 (COX2), as typical markers of the oxidative stress pathway.

**Figure 11:**
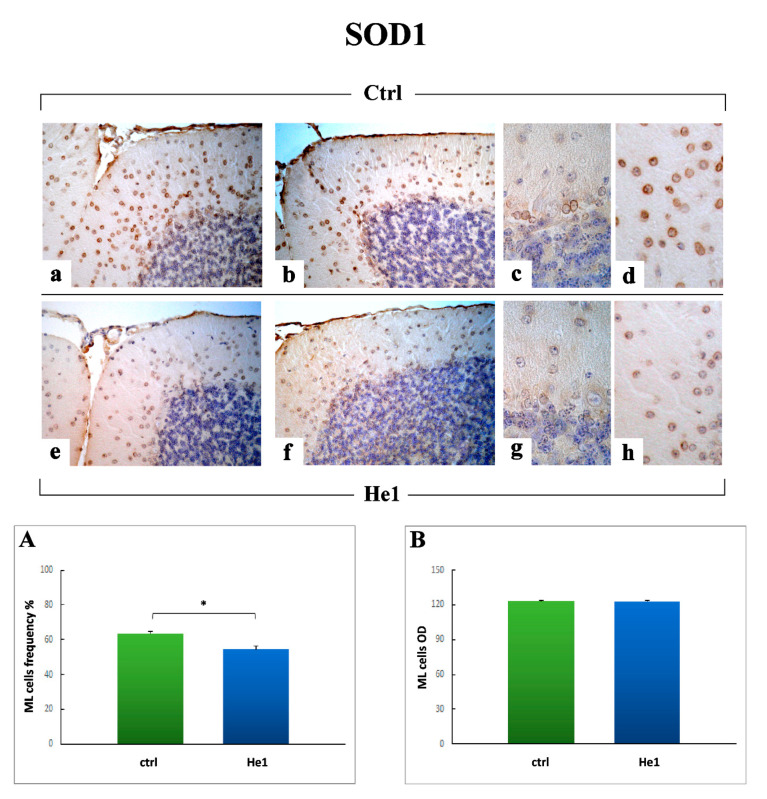
SOD1-Immunohistochemical labeling in untreated (**a**–**d**) and He1-treated mice (**e**–**h**). Light microscopy magnification: 40× (**a**,**b**,**e**,**f**); 100× (**c**,**d**,**g**,**h**). Panels (**A**,**B**) Histograms illustrating the quantitative measurement of SOD1 immunopositive cell frequency and OD, respectively. *p* values calculated by unpaired Student’s *t*-test: * *p* < 0.05.

The localization and expression of SOD1 revealed a widespread distribution both in untreated animals (Figure 11a–d) and He1 mice (Figure 11e–h); specifically, numerous SOD1-immunopositive cells were detected in the width of the ML (Figure 11d,h). Interestingly, some SOD1-immunopositive small cells lying just beneath the PC layer, possibly Lugaro cells, were identifiable in untreated mice only (Figure 11c). Notably, the quantitative analysis of SOD1 immunopositive cells frequency (54.44 ± 1.86 vs. 63.52 ± 1.26, *p* < 0.05) and OD (122.99 ± 0.68 vs. 123.15 ± 0.43) (Figure 11, Panels A and B, respectively), performed considering the ML layer, revealed a significant decrease in He1 mice compared to untreated animals.

**Figure 12:**
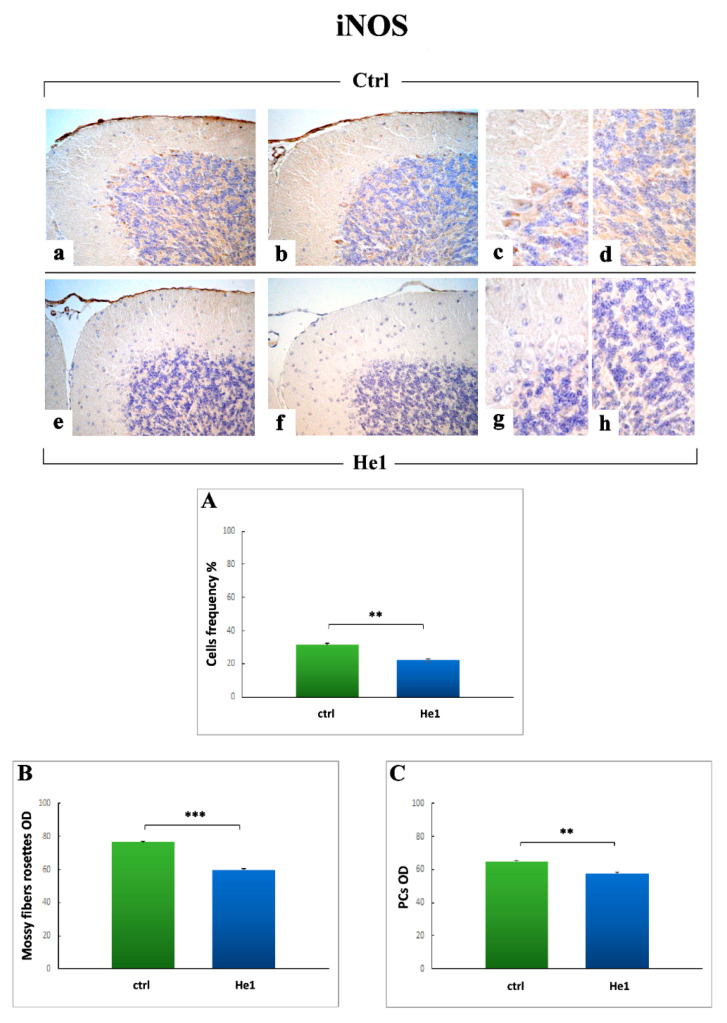
Immunohistochemical labeling for NOS2 in untreated animals (**a**–**d**) and He1 mice (**e**–**h**). Objective magnification: 40× (**a**,**b**,**e**,**f**); 100× (**c**,**d**,**g**,**h**). Panels (**A**–**C**) Histograms presenting the quantitative measurement of NOS2 immunopositive cell frequency, and OD evaluation for both mossy fibers rosettes as well as Purkinje neurons. *p* values calculated by unpaired Student’s *t*-test: ** *p* < 0.01, *** *p* < 0.001.

Concerning NOS2, a marked immunopositivity appeared evidently localized in the large soma of Purkinje neurons, both in untreated (Figure 12a–c) and He1 mice (Figure 12e–g). A clear immunopositivity was also detected in IGL, localized in the large mossy fiber rosettes (Figure 12d,h). Similarly to the observed SOD1 trend, NOS2-immunoreactivity, quantitatively measured in terms of cell frequency, i.e., immunopositive Purkinje neurons soma, significantly decrease in He1 treated animals compared to untreated animals (22.01 ± 1.07 vs. 31.31 ± 0.99, *p* < 0.01). (Figure 12, Panel A). Likewise, the analysis of NOS2-immunoreactivity OD, evaluated both at mossy fiber rosettes and Purkinje cell soma levels, significantly decrease in He1 mice compared to untreated ones (59.81 ± 0.91 vs. 76.52 ± 0.28, *p* < 0.001 and 57.66 ± 0.59 vs. 64.98 ± 0.62, *p* < 0.01, respectively) (Figure 12, Panels B and C).

**Figure 13:**
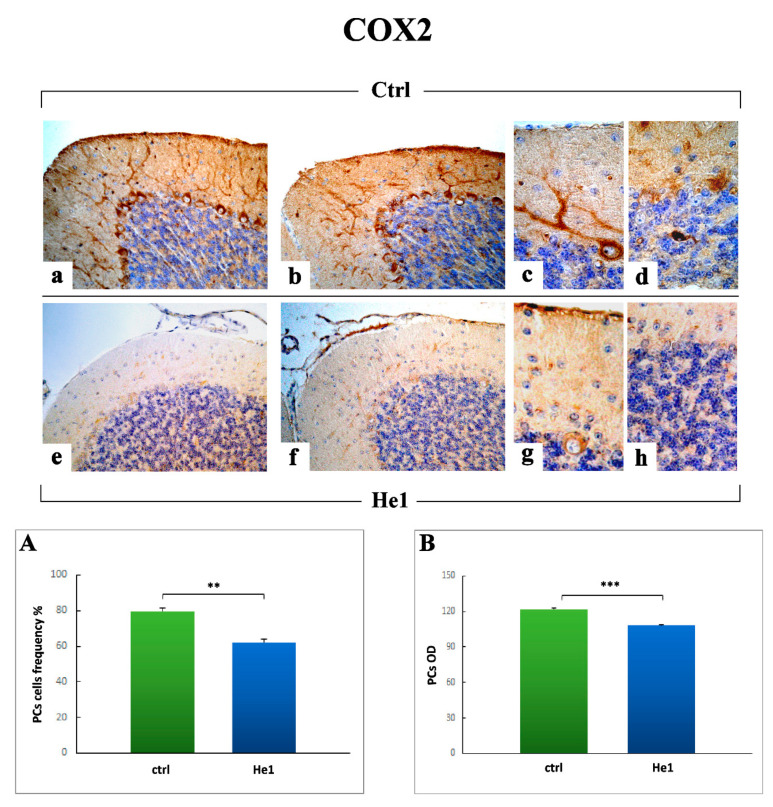
COX2 DAB-immunostaining reaction in untreated (**a**,**b**) and He1 mice (**c**,**d**). Light microscopy magnification: 40× (**a**,**c**); 100× (**b**,**d**). Panels (**A**,**B**) Histograms showing the quantitative determination of COX2 immunopositive cell frequency and OD. *p* values calculated by unpaired Student’s *t*-test: ** *p* < 0.01, *** *p* < 0.001.

Regarding COX2, a clear immunoreactivity was observed in the somas and main dendrites of Purkinje neurons, appearing more marked in untreated animals (Figure 13a,b) compared to He1 mice (Figure 13c,d). Notably, some heavily immunopositive Golgi cells were also observed in the IGL of untreated mice only. In accordance to the qualitative data, the successive quantitative analysis confirmed a significant decrease of COX2 immunoreactivity, evaluated in terms of both cell frequency and OD, in He1 mice compared to untreated animals (62.32 ± 1.67 vs. 79.67 ± 1.71, *p* < 0.01 and 108.65 ± 0.30 vs. 121.93 ± 0.49, *p* < 0.001, respectively. Figure 13, Panels A and B).

#### 2.3.5. Key Longevity Regulator: SIRT1

To further enhance the study of aging process and mechanisms, we finally investigated SIRT1, possessing a pivotal role impinging on ageing and lifespan, crucially affecting several activities in CNS neurons.

**Figure 14:**
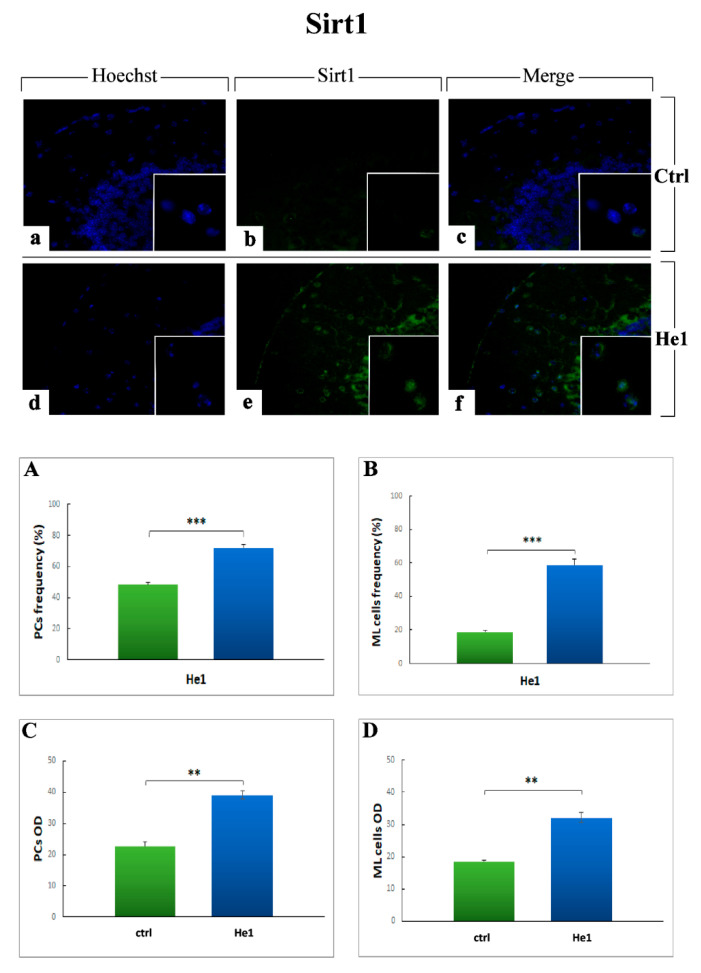
Immunofluorescence reaction for SIRT1 (green fluorescence) in untreated (**a**–**c**) and He1 mice (**d**–**f**). Nuclei were counterstained with Hoechst 33,258 (blue fluorescence). Objective magnification: 40× (**a**–**f**); 100× (*inserts*). Panels (**A**–**D**) Histograms showing quantitative analyses of SIRT1 immunopositive ML cell frequency and OD and immunoreactive PCs soma frequency and OD, respectively. *p* values calculated by unpaired Student’s *t*-test: ** *p* < 0.01, *** *p* < 0.001.

The evaluation of SIRT1 localization and expression showed a prevalent distribution in the ML and PCs layers, with a stronger immunolabeling in He1 mice compared to controls (Figure 14a–f); specifically, numerous SIRT1-immunopositive cells were detected in the ML thickness and the presence of several immunoreactive Purkinje cells soma was also observed (Figure 14b,c,e,f). Notably, the quantitative measurement of SIRT1 immunopositive ML cells frequency (59.00 ± 3.81 vs. 19.00 ± 1.08, *p* < 0.001) and OD (321.19 ± 15.48 vs. 185.69 ± 3.21, *p* < 0.01) demonstrated a significant increase in He1 treated mice compared to controls (Figure 14, Panels B and D). Concerning immunopositive PCs soma, the quantitative analyses established a significant increase of SIRT1 immunoreactivity, evaluated in terms of both cell frequency and OD, in He1 animals compared to untreated mice (72.32 ± 2.46 vs. 48.00 ± 1.44, *p* < 0.001 and 390.14 ± 13.34 vs. 226.00 ± 15.31, *p* < 0.01, respectively. Figure 14, Panels A and C).

## 3. Discussion

Literature evidence strongly supports that cerebellum, other than the cerebral cortex, is crucially implicated in age-related cognitive and motor declines. Moreover, several works in humans, non-human primates and rodents underscore the importance of investigating cerebellar subregions, as aging differentially impacts the cerebellar areas associated with cognitive and motor function [67]. Numerous findings further implicate the cerebellum in motor declines in older adults, also supporting the idea that cerebellar-prefrontal circuits may be especially important for motor and cognitive performance in older age. Thus, a clear pattern emerged, confirming that cerebellum is related to performance across a variety of motor and cognitive task domains in older individuals.

In previous works, we tuned a phenotypic and cognitive frailty index and we monitored frailty during physiological aging. Interestingly, locomotor decline preceded and was faster compared to cognitive decline. In particular, we focused on the effects of *H. erinaceus* extract on the partial recovery of recognition memory of novelty during physiological aging. In the present study, we focused on locomotor performance recovery in frail mice and we described, by means of different spontaneous tasks and by measuring different locomotor parameters, that *H. erinaceus* partially recovered frailty decline of locomotor performances. Notably, taken together, our data are in agreement with a double protective effect on both locomotor and cognitive decline of *H. erinaceus* during physiological aging in mice.

Cerebellum volumetric decrease may be a key factor contributing to the age-related declines seen in both motor and cognitive performances [58,59,68,69].

Thus, based on literature data, taking into consideration that (i) cerebellar subregions are distinctly related to performance in elderly, and (ii) the posterior vermis region of the cerebellum is particularly affected by ageing [59,70], we focused our study on neocerebellar lobules VI–VIII.

H&E and Nissl findings clearly evidenced an age-related alteration in untreated mice during physiological ageing, in which both the cerebellar volume and ML width were significantly reduced and about half of the PCs appeared shrunken. Notably, He1 animals exhibited a significantly greater cerebellar size and ML thickness, fewer altered PCs, paralleled by a significant reduction of fibrotic response, thus supporting a considerable protective role of He1 supplementation on aging in frail mice. Concerning PCs and ML, our data is in accordance with previous experimental evidence demonstrating that during normal aging, deafferentation of PCs leads to their progressive defoliation and, thus, to a thinner ML in rodent cerebellum [71,72].

Moreover, in line with our findings, age-dependent changes in cerebellar size have also been described in clinical and experimental research, suggesting a decrease in cerebellum size with aging [67,72,73,74,75].

Concerning the inflammatory pathway, our findings revealed a significant decrease of IL6 immunopositive cells and fibers (both in terms of frequency and OD) in He1 mice compared to untreated animals. Notably, this outcome was accompanied by a parallel significant reduction of both GFAP immunopositive fibers and astrocytes (density as well as OD) in He1 mice only. These data matched and appeared strictly linked, in that the observed IL6 immunopositive cells and fibers could be reasonably considered as the main constituents of the BG glia, with astrocytic cell bodies around the PCs and the radial fibers running the width of the ML, which also appeared immunoreactive for GFAP. In this view, we may hypothesize that the observed reactive gliosis could be a direct consequence of IL6 overexpression, known to arise with aging [60,61,76] or even that an opposite mechanism may occur.

Interestingly, recent data demonstrated that cerebellar reactive gliosis disrupts the spatial distribution of excitatory amino acids transporter 1 or glutamate aspartate transporter, resulting in an increase in extracellular glutamate concentrations and cytotoxicity. In addition, activated astrocytes and microglia have been demonstrated to release various proinflammatory molecules, including IL6 and tumor necrosis factor (TNF), implicated in neurodegeneration and negatively affecting Purkinje neuron function and survival [77].

Moreover, it has to be taken into consideration that glial reactivity increases with age and that hypertrophic, reactive astrocytes predominate in old brains, indicating the occurrence of a chronic neuroinflammation phenomena, the so-called “inflammaging” [78]. Moreover, complex changes in the immune system during aging were reported and referred to as immunosenescence, commonly accompanied by low-grade chronic inflammation thought to crucially contribute to neuroinflammation [79]. This latter aging hallmark is reported to have a dichotomous impact on glial activation, as these cells release pro- and anti-inflammatory cytokines and chemokines, antioxidants, free radicals, and neurotrophic factors, depending on the age and stimuli, even though the precise underlying mechanisms are still unknown. Furthermore, experimental studies in lab rodents revealed that astrocytes undergo age-dependent gene expression changes which contribute to synapse loss and neuroinflammation in a region-specific manner, thus revealing a selective vulnerability of some brain areas, e.g., cerebellum and hippocampus, to the aging process [80,81]. Recently, the involvement of neuroinflammation and gliosis in the pathogenesis of age-related and neurodegenerative diseases, e.g., Alzheimer, has been emphasized by compelling evidence from basic and clinical research studies [82,83].

In recent years, another assumption has gained particular attention hypothesizing the existence of a “molecular inflammatory theory of aging”, based on which the activation of redox-sensitive transcriptional factors by age-related oxidative stress causes the upregulation of proinflammatory gene expression. As a consequence, several proinflammatory molecules would be produced, leading to systemic and organ inflammation processes. During aging, this inflammatory cascade is particularly amplified, and has been linked with the role played by ROS, able to modulate various signals causing accelerated cellular senescence.

Accordingly, many reports documented the complex relationship between oxidative stress and inflammation, showing inflammation-mediated oxidative damage and remarking that oxidative stress may act as a critical mechanism linking inflammation, excessive extracellular matrix deposition and apoptosis. Nonetheless, whether the inflammatory or the oxidative stress response occurs firstly, still remains a highly debated question.

With regard to oxidative stress pathway, our findings demonstrated a marked reduction of both iNOS and COX2 immunopositivity in He1 mice only, paralleled by a concurrent slighter, but even significant, decrease of SOD1 immunoreactivity.

A bulk of literature undoubtedly proved that oxidative damage is involved in aging and age-associated cognitive impairment. In fact, during aging, neurons tend to gather impaired/aggregated proteins and damaged mitochondria, as a consequence of oxidative stress. The increased production of reactive oxygen species (ROS) together with the reduction in antioxidant scavengers represent the key players in this unbalanced process. Thus, a failure in the normal antioxidant defense mechanisms arises, which renders the aged brain more vulnerable to the consequences of oxidative stress [82,83]. Several experimental investigations demonstrated that longer-lived animals show reduced oxidative damage and/or increased resistance to oxidative stress, gained through dietary restriction or genetic manipulations in mice which increased lifespan [84]. In line with these latter, our present results supported the protective role of the oral supplementation with *H. erinaceus*, able to diminish expression levels of iNOS, COX2, and also SOD1, thus possibly increase lifespan or even ameliorate the quality of life together with locomotor performances.

Our data are in line with previous literature evidence demonstrating that natural extract-enriched diet reduced age-associated increases in COX-2 and iNOS expressions [85].

Our findings on inflammatory and oxidative stress pathways were further integrated by the data on HIF1α, known to play a crucial role in aging-related pathology, including vascular diseases [64]. Emerging evidence has identified HIF1α as a critical transcription factor with an essential role in regulating cellular senescence associated with aging, which is in turn associated with alterations in HIF1α production and function [64,86]. Certainly, it has to be underlined that published data on HIF1α often appear contradictory, since its upregulation resulted either protective or detrimental, depending on the measurement conditions. For example, increased HIF1α levels typically determined in age-related and neurodegenerative pathologies, e.g., Alzheimer’s and Parkinson’s diseases, have been indicated as a clear sign of advanced disease progression. Differently, other literature evidence implied the attenuated activity of HIF1 in senescent tissues as a key factor in the decreased ability to respond to hypoxic stress [87].

Our results evidenced a specific HIF1α immunoreactivity in vascular endothelial cells, in the width of the entire cerebellar cortex. Notably, we measured a significant decrease of both blood vessels area and OD in He1 mice compared to untreated animals. These data could be related to the role of HIF1α as a regulator of angiogenesis and inflammation in aging disorders, by promoting pro-inflammatory cytokine expression and, consequently, inflammatory cells recruitment, often associated with increased VEGF levels. In particular, based on the notion that HIF1α increases with age, perhaps due to enhanced hypoxia and oxidative stress, also acting as a master regulator in vascular aging [64,87], our results supported a positive protective action of *H. erinaceus*, able to maintain HIF1α levels stable over time, or even to decrease them. These protein levels could be linked to the reduction of both inflammation and oxidative stress, above reported in He1 mice only, as well as to a decrease of hypoxic conditions due to medicinal mushroom action, capable of mitigating age-associated alterations.

Concerning VEGF, its role in neural protection has recently gained much interest. In fact, other than vasculo-angiogenic properties, recent findings pointed out VEGF’s role as a neurogenic, neurotrophic and neuroprotective factor in the nervous system, particularly implicated as a key player in the cerebellum [65]. Specifically, in the cerebellum, VEGF is extensively expressed in neurons, glia and endothelial cells with higher levels in Purkinje neurons. Notably, in adult CNS, VEGF is able to arouse neuronal recovery from injuries, e.g., strokes or epilepsy seizures, while, on the contrary, VEGF low level or depletion can negatively influence Purkinje neurons survival, facilitating neuronal injury outcome [65,88]. Interestingly, the role of VEGF in the age-related neurodegenerative diseases, e.g., Alzheimer’s disease (AD) dementia, is still debated and controversial since diverse literature evidence exists for both up- and downregulation of VEGF in the brain. Nonetheless, growing evidence supports a neuroprotective role played by VEGF family protein expression associates in cognitive aging and AD pathology [89].

Our present results revealed a significant increase of VEGF expression levels, mainly localized in Purkinje cells soma and mossy fibers rosettes, in He1 treated mice only, evaluated in terms of both cell frequency and OD, while a complete lack of immunoreactivity was evidenced at blood vessels level. These data corroborate the neuroprotective action of *H. erinaceus* standardized extracts, whose intake led to a restoration/enhancement of VEGF expression levels, supporting the role of VEGF as an important nootropic factor capable of improving neurotrophic support to cerebellar neurons, thus ensuring or at least bettering their survival in aged-related conditions.

Finally, our data established that the oral supplementation with *H. erinaceus* was able to increase SIRT1 expression levels, possibly improving animal lifespan, also ameliorating both quality of life as well as locomotor performances.

SIRT1 is known to be strongly implicated in the aging process of diverse organisms, including mammals, based on its ability to integrate multiple signaling and transcriptional pathways, in which SIRT1 participates to regulate cellular senescence, metabolic disorders, autophagy, DNA damage and mitochondrial dysfunction, all of which are hallmarks of aging [90,91,92]. SIRT1 role as longevity regulator is well-known, since it acts as a key molecule in neuronal plasticity, cognitive functions, as well as protection against ageing-associated neuronal degeneration and cognitive decline [92]. In accordance with previous studies demonstrating that SIRT1 is associated with lifespan extension and other antiaging effects, specifically broadening mice life expectancy when overexpressed in the brain [92,93], our results led us to reasonably hypothesize that He1 supplementation was able to activate an antioxidant mechanism by increasing the expression of SIRT1, which in turn decreases ROS levels, inhibiting apoptosis and promoting cell survival and neuroprotection. Notably, we may suppose that *H. erinaceus* oral supplementation could activate a stabilization mechanism in the regulation of the protein homeostasis of SIRT1 promoting healthy ageing.

**Figure 15:**
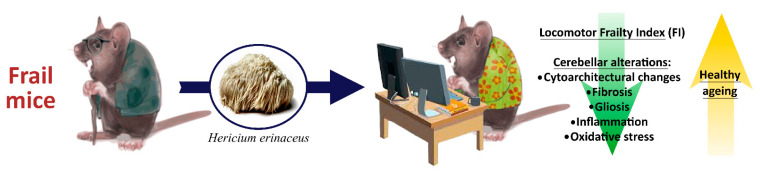
Schematic illustration summarizing main findings and the take-home message of the manuscript.

In summary, our present findings clearly supported a neuroprotective action of *H. erinaceus* supplementation, able to ameliorate the cerebellar condition in aged frail mice compared to untreated animals (Figure 15). Specifically, our data mainly evidenced a bettering of the gross anatomy and cytoarchitectural situation, with a clear reduction of inflammation, gliosis, and oxidative stress, accompanied by (i) increase of neuroprotection and (ii) enhancement of a master key longevity regulator, paralleled by improvement of locomotor performances (Figure 15). Therefore, it is reasonable to believe that the bioactive metabolite array contained in the *H. erinaceus* significantly impacted on cellular mechanisms and molecular pathways typically linked to age-related neurodegeneration and locomotor impairments. Moreover, cerebellum, other than prefrontal cortex, appeared as a good predictor of locomotor performance declines during aging, suggesting that forthcoming investigations, focusing on this area and its relationship with age-related disorders pathogenesis, would be especially enlightening.

The present findings are consistent with the well-established role of *H. erinaceus* as the “choice” medicinal mushroom capable of (i) promoting positive brain and nerve health-related activities by inducing the nerve growth factor from its bioactive ingredient [94]; (ii) impacting on inflammation typically linked to age-related neurodegenerative disease, i.e., ischemic stroke, Parkinson’s and Alzheimer disease, mainly acting in specific brain areas, i.e., cerebellum and cortex [32,94,95]; (iii) minimizing the oxidative stress-related consequences characteristics of brain aging and neurodegenerative disorders [83]; (iv) reducing anxiety and depressive behaviors by promoting hippocampal neurogenesis in the adult mouse [96].

Finally, it has to be highlighted that our current data are in line, and even strengthened, our previous findings demonstrating that dietary supplementation with *H. erinaceus* was effective at (i) increasing hippocampal neurotransmission, locomotor performances and recognition memory in wild-type mice [50,51], and (ii) improving recognition memory in frail mice during aging, also inducing hippocampal and cerebellar neurogenesis [14].

For completeness, it has however to be mentioned that in the present study we evaluated the effects of a particular *H. erinaceus* strain extract, characterized by the presence of different metabolites (i.e., Erinacine A, Hericenone C, Hericenone D, and ergothioneine), precisely quantified using HPLC-UV-ESI/MS by comparison with specific standard solutions. During our next investigations, it would be advantageous either to perform a further in-depth chemical analysis employing additional standards to identify other unknown metabolites contained in the currently employed *He1* extract or to assess the potential nootropic action of other metabolite-enriched *H. erinaceus* strains. Moreover, our results could be replicated and thus further confirmed on a larger set of animals which could be randomized into different experimental groups, also widening the exploited age-related markers.

As a final point, it has to be underscored that we presently employed both open field and emergence tests for measuring the extent of locomotor frailty in order to reach a translational approach with human test, since the gait speed is one of the accepted clinical parameters used in Fried’s phenotype model for frailty in older adults. This simplified, noninvasive method allows us to monitor the development of frailty during mice aging both as phenotypic and cognitive frailty. Nonetheless, further investigations would also include additional locomotor and performance tests, including for example grip strength, rotarod test or voluntary wheel running, paralleled by cognitive tests, assessing recognition memory, e.g., novel object recognition and object location tasks [51], that are used to monitor cognitive frailty development during ageing. These future data would further strengthen the current results also in accordance with literature evidence, underlining that frailty index score needs to be tuned by a number of different locomotor parameters since it has been recognized that several task parameters need to be recorded to validate and give interpretation of behavioral experiment data.

## 4. Materials and Methods

### 4.1. Animals and Treatments

**Figure 16:**
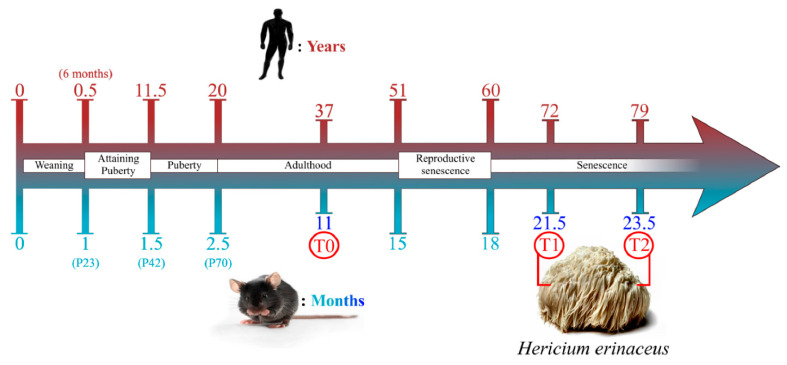
Schematic drawing summarizing experimental time course, chosen timepoints and comparison between men and mice age during their life span (modified from [14]).

Fifteen C57BL-6J wild-type male mice were purchased from Charles River Italia (Calco, Italy). The pathogen-free mice were acclimatized at least 2 weeks before conducting the experiments. Mice were maintained in the Animal Care Facility of University of Pavia at 21 ± 2 °C, with humidity at 50 ± 10%, and on a 12 h light/dark cycle. Water and food were provided ad libitum. The experimental procedures were carried out in accordance with the guidelines laid out by the institution’s animal welfare committee, the Ethics Committee of Pavia University (Ministry of Health, License number 774/2016-PR). Behavioral tests experiments were performed at three different animal ages: 11 (T0, recruitment time, including in adulthood), 21.5 (T1, senescence) and 23.5 (T2, senescence) months old. The seven frailest mice (He1 treated group), as assessed by locomotor frailty index, were supplemented for two months (starting from T1) with a drink made with a blend of *H. erinaceus* sphorophore and mycelium ethanol extracts solubilized in water (Figure 16), with the purpose to administer 1 mg supplement/mouse per day [14]. This amount was selected to mimic the oral supplementation in humans (about 1 g/day).

### 4.2. Behavioral Tests and Locomotor Frailty Index

In vivo experiments were performed for investigating locomotor abilities of mice. For all experiments, researchers were blinded to the group assignment (untreated and He1 treated mice). Mice performances were measured by SMART video tracking system (2 Biological Instruments, Besozzo, Varese, Italy) and Sony CCD color video camera (PAL). All mice, at different experimental times, performed two spontaneous behavioral tests: open arena and emergence tasks. In the open arena test, mice were left free to explore an empty arena of 63 × 42 cm. During the emergence test, mice were placed in a familiar cage with a hole through which they can emerge in a larger arena without walls [51]. Both tests lasted 8 min; mean speed (cm/s), resting time (s), and max speed (cm/s) were evaluated. For each parameter the corresponding locomotor frailty index (FI) was calculated by using the following formula [14]:

FI = (Value-Mean Value at T0)/(SD at T0) × 0.25

Averaging the different locomotor FIs for the two selected parameters (mean speed and resting time), we obtained an overall Locomotor FI, which described the overall locomotor decline during senescence.

### 4.3. H. erinaceus

The He1 (strain 1 of *H. erinaceus*) was isolated from a wildtype sporophore collected in 2013 in Siena province (Tuscany, Italy) from a Quercus ilex [97]. The sporophore was aseptically cut and placed in 2% malt extract agar as a culture medium (MEA, Biokar Diagnostics) [14]. The isolated strain was further maintained in the Fungal Research Culture Collection of Pavia University (MicUNIPV).

#### 4.3.1. Extraction Procedures

Based on the lack of formerly published standardized extraction protocol for the ergothioneine (ET), we adopted the method described by Cesaroni et al. [97,98] as a starting point, and then rearranged it with the extraction procedure already reported in Corana et al. [52] and Ratto et al. [14] for erinacines and hericenones, metabolites present in *H. erinaceus*. In detail, 1 g of lyophilized mycelium or sporophore of He1 was blended with 10 mL of ethanol 70%, and left in the thermostat overnight at 50 C. Before withdrawing, the material was stirred for one hour and was centrifuged at 4000 rpm for three minutes. The supernatant was stored at 20 °C. The detailed procedures were previously described by Ratto et al., Lee et al., and Gerbec et al. [14,52,99,100].

#### 4.3.2. HPLC-UV-ESI/MS Method

HPLC-UV-ESI/MS analyses were carried out as previously reported [14].

In order to identify and measure the amount of ET, we analyzed the mycelium and sporophore extracts of He1 using HPLC-UV-ESI/MS, by comparing to ET standard. L-(+)-Ergothioneine (ET; E7521-5MG, Sigma Aldrich, Milan, Italy) was used as standard. The ET calibration curve was constructed by injecting standard mixture solutions at five concentrations (10, 70, 150, 350 μg/mL). Each concentration was analyzed in triplicate.

### 4.4. Tissue Sampling, Histology, Immunohistochemical and Immunofluorescence Evaluations

For each treatment, cerebellar tissues were processed for the following morphological and histochemical evaluations.

#### 4.4.1. Cerebellar Specimens Preparation

At the age of 23.5 months (T2), mice were anesthetized by isoflurane inhalation (Aldrich, Milwaukee, WI, USA) before decapitation. Cerebella were immediately excised as previously described [101], washed in 0.9% NaCl, and fixed by immersion for 48 h at room temperature in Carnoy’s solution (6 absolute ethanol/3 chloroform/1 acetic acid). Tissues were then kept in absolute ethanol for one hour, followed by acetone, and finally embedded in Paraplast X-TRA (Sigma Aldrich, Milan, Italy). Eight micron-thick sections, collected on silane-coated slides, of cerebellar vermis were cut in the sagittal plane.

#### 4.4.2. Post-Embedding Cerebellar Volume Estimation

The Cavalieri method was employed, following procedures previously described [75,102]. Briefly, sections were traced on the Nissl-stained sections at 4× magnification to estimate the surface area. No discrimination among separate layers was made due to sections thickness. Then, the following formula was used to estimate the volume of the whole cerebellum:

Volume = P ∗ T ∗ A(p)

P was the sum of the points counted in all sections, T was the thickness of sections (8 µm), and A(p) was the area associated with each point; [100 µm/40]^2^ (where 4× was the magnification and 100 µm was the distance between each point).

#### 4.4.3. H&E and Nissl Staining

Subsequently, to overall evaluate structural changes and neuronal cytoarchitecture by light microscopy, H&E and Nissl staining were performed as previously described [103,104,105]. The slides were then observed and scored with a bright-field Zeiss Axioscop Plus 612 microscope. Specifically, 5 slides (about 20 randomized sections) per animal were analyzed; 5 microscopic fields were examined in each section for each mouse per time/condition, with the operator blinded to the experimental condition. The images were recorded with an Olympus Camedia C-5050 digital camera and stored on a PC running Olympus software.

#### 4.4.4. Picrosirius Red (PSR) Staining

Serial tissue sections were stained for 1 h with a PicroSirius red (PSR) solution (0.1% of Sirius red in saturated aqueous picric acid), followed by a wash in 5% acidified water [106,107], for collagen staining. Lastly, the sections were dehydrated in ethanol, cleared in xylene, and finally mounted in Eukitt (Kindler, Freiburg, Germany).

#### 4.4.5. Immunohistochemistry: Light and Fluorescence Microscopy Assessment

Immunohistochemical reactions were carried out simultaneously on slides from different experimental groups to avoid possible staining differences due to small changes in the procedures. Immunohistochemical and immunofluorescence procedures were performed using commercial antibodies on murine cerebellar specimens, to investigate expression and distribution of specific markers representative of inflammation, reactive gliosis, oxidative stress and age-related mechanisms: (i) Interleukin-6 (IL6), (ii) Glial fibrillary acidic protein (GFAP), (iii) hypoxia-inducible factor (HIF1α), (iv) Cu–Zn superoxide dismutase-1 (SOD1), (v) nitric oxide synthase 2 (NOS2), and (vi) cyclo-oxygenase-2 (COX2), (vii) Sirtuin 1 (SIRT1), and (viii) vascular endothelial growth factor (VEGF).

Cerebellar sections of untreated and He1 mice were incubated overnight at room temperature in a dark moist chamber with selected monoclonal and polyclonal primary antibodies (Table 2) diluted in PBS. In detail, GFAP immunostaining was employed to mark specifically Bergmann glia [108,109]; IL6 has been investigated as valuable inflammation marker [61,110]; SOD1, NOS2 and COX2 were examined being specific molecules essentially involved in oxidative stress pathway [15,111,112,113,114,115]; SIRT1 was assessed for its pivotal role that impinges on cellular senescence and lifespan [90,92,116]; VEGF was considered possessing broad-ranging functions, both in vascular system and CNS [65].

Concerning immunohistochemical reactions detected using brightfield microscopy, proper biotinylated secondary antibodies (Table 2) and an avidin biotinylated horseradish peroxidase complex (Vector Laboratories, Burlingame, CA, USA) were used to reveal the sites of antigen/antibody interaction. The 3,3′-diaminobenzidine tetrahydrochloride peroxidase substrate (Sigma, St. Louis, MO, USA) was used as the chromogen. The nuclear counterstaining was achieved by employing Carazzi’s Haematoxylin. Then, the sections were dehydrated in ethanol, cleared in xylene, and finally mounted in Eukitt (Kindler, Freiburg, Germany). As negative untreated, some sections were incubated with PBS in the absence of the primary antibodies: no immunoreactivity was observed in this condition.

Regarding immunofluorescence reactions, after washing in phosphate buffer saline (PBS), sections were incubated for one hour with the secondary antibody Alexa Fluor 488-conjugated anti-rabbit (1:100, Thermo Fisher scientific, Invitrogen, Waltham, MA, USA) in a dark moist chamber. Then the nuclei were counterstained for 10 min with 0.1 μg/mL Hoechst 33,258 (Sigma Aldrich, Milan, Italy). After PBS washing, coverslips were mounted in a drop of Mowiol (Calbiochem, San Diego, CA, USA).

#### 4.4.6. Histochemical, Immunohistochemical and Immunofluorescence Evaluations

Regarding brightfield microscopy, the sections were observed with an Olympus BX51 optical microscope (model BX51TF). The images were acquired with an Olympus CAMEDIA C4040ZOOM camera.

For each selected marker, 5 slides (about 20 sections) per animal were analyzed. Cerebellar specimens with different immunolabeling extent were considered in both experimental groups. The figures show the most representative changes for each immunohistochemical reaction.

Histochemical and immunohistochemical labeling extent was evaluated on acquired digitized section images under exposure time avoiding any pixel saturation effect. The labeling intensity was measured utilizing densitometric analysis (Image-J 1.48i; NIH, Bethesda, MA, USA). Firstly, the color of images was inverted to obtain a positive signal lighter on a dark background (instead of the immunoperoxidase staining results), thus correlating the intensifying of immunopositivity with the optical density values increasing calculated by the software (expressed as mean of light intensity). The mask shape was adjusted depending on the spatial distribution, signal localization, different layer and cell types/fibers of the cerebellar specimens under measurement (using the polygon selection tool, to ensure the punctual evaluation of the positivity area only); the labeling was measured as the mean intensity value over the area. The immunocytochemical intensity, indicated as optical density (OD), was evaluated in 3 randomized images/section (making at least 10 measurements/image) per 5 slides/animal from each experimental group, with the operator blinded to the experimental condition. Results were recorded on Microsoft Office Excel Software spreadsheets and the analysis was achieved using the ImageJ software.

The following further measurements were performed: (i) ML thickness (by using a 40× objective on H&E-stained slides) in µm; (ii) GFAP-immunopositive Bergmann glia area in µm^2^/whole ML area in µm^2^, (iii) GFAP-immunopositive cells density count (number of immunopositive cells/area in mm^2^) and (iv) HIF1α-immunopositive blood vessels area in µm^2^/whole area in µm^2^.

Concerning fluorescence microscopy, sections were observed with an Olympus BX51 optical microscope equipped with a 100-W mercury lamp used under the following conditions: 330–385 nm excitation filter (excf), 400 nm dichroic mirror (dm), and 420 nm barrier filter (bf) for Hoechst 33,258; 450–480 nm excf, 500 nm dm and 515 nm bf for the fluorescence of Alexa 488. Images were recorded with an Olympus MagnaFire camera and results were processed with the Olympus Cell F software.

Immunofluorescence quantification was performed by calculating frequency (percentage ratio) and optical density (OD) of SIRT1 immunopositive cells on a total number (about 300 cells) for each animal per experimental condition, in a minimum of 10 randomly selected high-power microscopic fields, with the operator blinded to the experimental condition.

### 4.5. Statistical Analysis

Data were reported as mean ± standard error of the mean (SEM). We performed Bartlett and Shapiro Wilk Tests to establish and confirm the normality of parameters. To verify statistically significant differences, we used a two-way Anova followed by Bonferroni’s *post-hoc* test for the *H. erinaceus* supplementation effect in vivo. The statistical analysis for histochemical and immunohistochemical evaluations was carried out using an unpaired Student’s *t*-test. The differences were considered statistically significant for *p* < 0.05 (*), *p* < 0.01 (**), and *p* < 0.001 (***). Statistical analyses were performed with GraphPad Prism 7.0 software (GraphPad Software Inc., La Jolla, CA, USA).

## 5. Conclusions

In conclusion, our results provide experimental evidence that *H. erinaceus* may supply neuroprotective metabolites to be used as valuable, effective candidates for treating and preventing age-related neurodegenerative diseases. Altogether, our results support the potential feasibility of non-pharmacological approaches, including dietary supplementations using medicinal mushrooms extracts, as promising adjuvant therapies to be associated with conventional geriatric treatments.

## Figures and Tables

**Table 1:** Ergothioneine molecular formula, molecular weight, chemical structure, characteristic and fragment ions, and mycelium and sporophore amounts are summarized.

Molecular Formula	Molecular Weight (g/moL)	Chemical Structure	ESI/MS Characteristic Ions (*m*/*z*)	ESI-MS/MS Fragment Ions (*m*/*z*)	Content (mg/g)
C9H15N3O2S	229.3	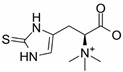	230 [M+H]+ 252 [M+Na]+ 268 [M+K]+	186 [M+H-CO_2_]+ 127 [M+H-CO_2_-(CH3)3N]+	0.58 (mycelium) 0.34 (sporophore)

**Table 2:** Primary/secondary antibodies employed for immunocytochemical/immunofluorescence experimental procedures.

	Antigen	Immunogen	Manufacturer, Species, Mono-Polyclonal, Catalogue or Lot No., RRID	Diluted Used
**Primary antibodies**	*Anti-Interleukin-6 (M-19)*	Purified antibody raised against a peptide mapping at the C-terminus of murine IL6	Santa Cruz Biotechnology (Santa Cruz, CA, USA), Goat polyclonal IgG, Cat# sc-1265, RRID: AB_2127470	1:100
	*Anti-Glial fibrillary acidic protein (C-19)*	Purified antibody raised against a peptide mapping at the C-terminus of GFAP of human origin	Santa Cruz Biotechnology (Santa Cruz, CA, USA), Goat polyclonal IgG, Cat# sc-6170, RRID: AB_641021	1:100
	*Anti-Hypoxia-inducible factors-1α (H1α 67)*	Purified antibody raised against amino acids 432–528 of HIF-1α of human origin	Santa Cruz Biotechnology (Santa Cruz, CA, USA), Mouse monoclonal IgG, Cat# sc- 53546, RRID: AB_629639	1:100
	*Anti-Superoxide Dismutase-1 (FL-154)*	Purified antibody raised against amino acids 1–154 representing full-length human SOD-1	Santa Cruz Biotechnology (Santa Cruz, CA, USA), Rabbit polyclonal IgG, Cat# sc-11407, RRID: AB_2193779	1:100
	*Anti-Nitric Oxide Synthases-2 (M19)*	Purified antibody raised against a peptide mapping at the C-terminus of murine NOS-2	Santa Cruz Biotechnology (Santa Cruz, CA, USA), Rabbit polyclonal IgG, Cat# sc-650, RRID: AB_631831	1:100
	*Anti-Cyclooxygenase-2 (M-19)*	Purified antibody raised against a peptide mapping at the C-terminus of murine COX-2	Santa Cruz Biotechnology (Santa Cruz, CA, USA), Goat polyclonal IgG, Cat# sc-1747, RRID: AB_2084976	1:100
	*Anti-Sirtuin1*	Purified antibody raised against amino acids 722–737 of murine SIRT1 with a C-terminal added lysine	Abcam (Cambridge, MA, USA), Rabbit polyclonal IgG, Cat# ab12193, RRID: AB_298923	1:100
	*Anti-VEGF*	Purified antibody raised against a peptide corresponding to aa 1–140 of VEGF of human origin.	Santa Cruz Biotechnology (Santa Cruz, CA, USA), Rabbit polyclonal IgG, Cat# sc-507, RRID: AB_2212666	1:100
**Secondary antibodies**	*Biotinylated goat anti-rabbit IgG*	Gamma immunoglobulins	Vector Laboratories (Burlingame, CA, USA), Goat, lot# PK-6101, RRID: AB_2336820	1:200
	*Biotinylated rabbit anti-goat IgG*	Gamma immunoglobulins	Vector Laboratories (Burlingame, CA, USA), Rabbit, Cat# PK-6105, RRID: AB_2336824	1:200
	*Goat anti-Rabbit IgG (H+L) Cross-Adsorbed Secondary Antibody, Alexa Fluor 488*	Gamma Immunoglobulins (Heavy and Light chains)	Thermo Fisher scientific, Invitrogen (Waltham, MA USA), Cat# A-11008, RRID: AB_143165	1:100

## Data Availability

Not applicable.
